# Mating success follows duet dancing in the Java sparrow

**DOI:** 10.1371/journal.pone.0172655

**Published:** 2017-03-08

**Authors:** Masayo Soma, Midori Iwama

**Affiliations:** 1 Behavioral Neurobiology Group, Department of Biology, Faculty of Science, Hokkaido University, Kita 10, Nishi 8, Kita-ku, Sapporo, Hokkaido, Japan; 2 Behavioral Neurobiology Group, Biosystems Science Course, The Graduate School of Life Science, Hokkaido University, Kita 10, Nishi 8, Kita-ku, Sapporo, Hokkaido, Japan; University of Arkansas, UNITED STATES

## Abstract

Mutual interactions between sexes have multiple signalling functions. Duet singing in songbirds is related to mutual mate guarding, joint resource defence, and signalling commitment. Coordinated visual displays of mating pairs are thought to perform similar functions, but are less well understood. The current study evaluated mutual interactions in an Estrildid species to explore the relative importance of duet dancing and male singing in mating success of pairs in a first encounter. When Java sparrows (*Lonchura oryzivora*) court prospective mates, only males sing. However, both males and females perform courtship dances, often in a duet-like manner. These dances are typically terminated by female copulation solicitation displays (CSDs). In the current study, we observed higher mating success when courtship dances were mutually exchanged, and when males sang. However, the sex initiating the courtship did not affect mating success. Most females produced CSDs after duet dancing but before hearing the entire song, indicating that duet dancing played a crucial role in mating. This finding highlights an unexplored aspect of duetting behaviour in the process of mutual mate choice. These results conflict with the majority of past songbird research, which has interpreted songs as primary behavioural sexual signals.

## Introduction

Bird song is considered a classic example of a sexually selected behaviour, and is related to reproductive success [1–4]. Visual courtship displays in birds are thought to play a similar role in mating, but, unlike birdsong, there is a lack of empirical evidence regarding the effects of within- and between- individual differences in visual displays on reproductive outcomes [5]. Moreover, while some avian species exhibit courtship displays that include assemblages of vocalisation and body movements [6–9], far less attention has been paid to the relative importance of auditory and visual/gestural components of multimodal signals as sexual communication.

Estrildid finches have courtship displays that include song and dance (e.g., [9–13]). Male song and dance is invariably exhibited in courtship, but a high degree of interspecific diversity has been found in the presence or absence of female song and dance [14,15]. Moreover, a previous phylogenetic comparative study [15] reported that presence of female song was unrelated to the presence of female dance, indicating that the two traits evolved independently. This finding raises questions about the selective forces influencing the evolution of the two traits, highlighting the importance of examining the ways that multiple traits function as sexual communication signals [16,17]. Sexual signals that are shared between males and females, including ornamental plumage, song, and dance, are generally considered to be involved in mutual mate choice, or intrasexual competition [18,19]. However, the courtship behaviours of Estrildidae are not well understood in this paradigm.

Among birds, dynamic sexual signals are sometimes mutually exchanged between the sexes, a phenomenon commonly referred to as a duet. Coordinated duet singing performed by mated pairs is thought to be involved in mutual mate guarding, joint resource defence, and the signalling of commitment [20,21]. For example, several studies have reported that Australian magpie-lark (*Grallina cyanoleuca*) pairs that had been in longer partnerships produced more highly coordinated duet songs that served as effective territorial displays to rivals [22,23]. In addition, long-term canebrake wren (*Thryothorus modestus zeledoni*) pairs have been reported to sing duets with more consistent repertoires [24]. In a well-studied Estrildid, the zebra finch, mated pairs exhibit duets of calls at the nest after separation, which may be a mechanism for pair-bond maintenance [25]. However, although the blue-capped cordon-bleu (*Uraeginthus cyanocephalus*) is an Estrildid species known for female dance and song, very little duet dancing or singing has been observed in the species (pers. obs., but also see [11–13]). Whereas most previous duet research has focused on vocalisation, one recent study revealed that audio-visual signals performed by pairs of magpie-larks in duet played a similar role [26]. Thus, many previous studies of duet behaviour have indicated that pair-bond duration and strength can contribute to the performance of precisely coordinated displays of already-mated pairs, suggesting that such displays are effective for intra- or inter-pair communication [20].

It remains unclear how duet vocalisations are involved in earlier stages of social interactions between potential mating partners, such as the initiation of partnerships, and mate choice. In the canary-winged parakeet (*Brotogeris versicolurus*), duet singing has been observed to precede or coincide with pair establishment [27], but similar empirical evidence in other species is scarce. Some previous studies have suggested that visual displays exchanged and coordinated between males and females might be involved in pair courtship, playing a role in mutual mate choice or mate assessment in non-passerine species [28–30]. Duet singing and mutual dancing in birds share several characteristics, both involving joint performance of a male and a female, often with finely tuned temporal coordination (e.g., synchronisation, or antiphony/taking turns). However, the question of whether audio and visual displays have similar functions remains contentious [31,32].

**Fig 1:**
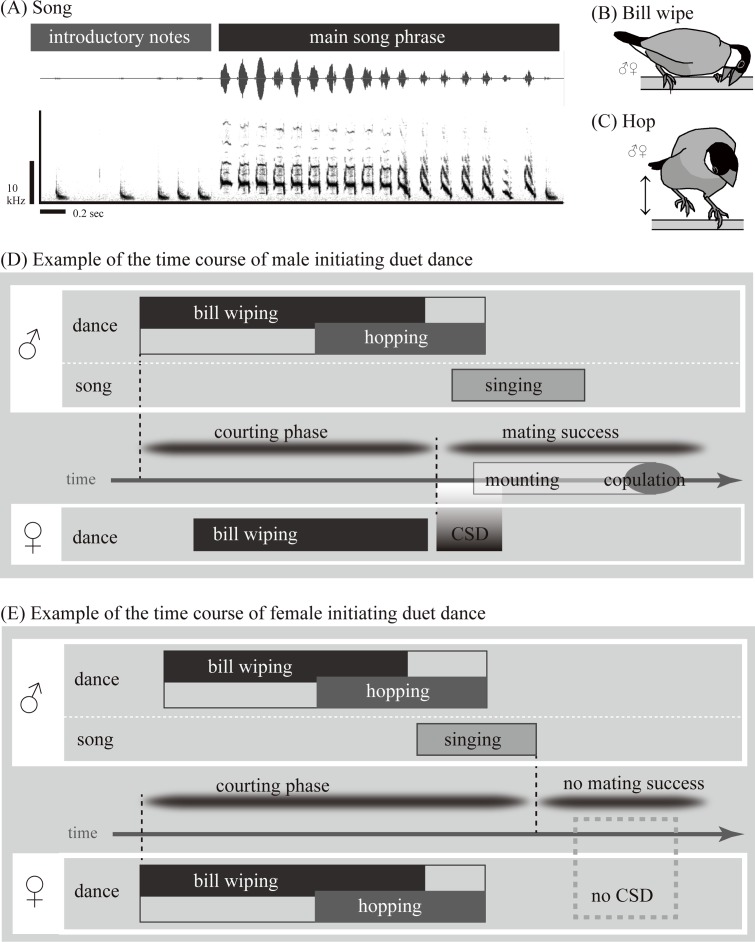
Schematic view of the Java sparrow courtship. Courtship of the Java sparrow includes male song and dancing by both sexes. (A) Songs are composed of introductory notes, sparse repetition of single notes with low amplitude, and a main song phrase that is characterised by a variety of notes. (B, C) Dance includes bill wiping and hopping. (D, E) Courtship can be initiated by either a male or a female, often followed by dance response of the partner bird. When any dance components (bill wiping or hopping) were mutually exchanged, the behaviour was considered duet dancing. The courting phase was considered to span from the start of the courtship until female CSDs or until the end of courting behaviours in cases without CSDs. Mating success was determined based on the presence of CSD, mounting and copulation that followed the courting phase.

The current study examined the Java sparrow (*Lonchura oryzivora*), a monogamous and sexually monochromatic songbird belonging to the Estrildidae family. In general, Estrildid finches have a highly gregarious nature without territories, and their songs are only used for courtship and not territorial defence [14,33]. Only male Java sparrows have been observed to produce songs, whereas both males and females perform courtship dances that are often mutually exchanged in a duet-like manner. These dances are typically terminated by females performing a copulation solicitation display (CSD), followed by copulation [14,33] (Fig 1; S1–S3 Movies). These behavioural features make the Java sparrow a suitable species for empirically testing the role of duet dancing, and investigating its involvement in mutual courtship preceding pair establishment. Thus, we examined the relative importance of song and dance in this species, in which dance is shared between the sexes but song is not. We observed behavioural interactions between males and females upon first meeting, and examined which behaviours were essential for mating success, based on the occurrence of CSD, mounting, and copulation.

## Methods

### Ethics statement

This study was conducted with approval from the Institutional Animal Care and Use Committee of the National University Corporation at Hokkaido University (No. 11–0028) in accordance with Hokkaido University Regulations of Animal Experimentation. During the study, stress was minimised and all birds were cared for and treated appropriately, in accordance with the Guidelines for Proper Conduct of Animal Experiments from the Science Council of Japan and the Guidelines for Ethological Studies from the Japan Ethological Society. After the study, birds were used either for other experiments or for breeding purposes.

### Study species

Java sparrows are native to Java and Bali in Indonesia, but are also popular as pets, and hence their display behaviours are well-described [14,33]. When Java sparrows court prospective mates, either a male or a female starts by bill wiping and hopping (Fig 1), which are often followed by male singing and female CSDs (S1–S3 Movies) [14,33]. The two main behavioural elements of courtship dance (hopping and bill wiping) are ritualised, and can be distinguished from non-courtship behaviours [14,33].

Because Java sparrows are less-well studied and endangered in their natural habitat, we lack information about their breeding ecology, including pair-bond tenure, and the rate of extra-pair copulation. According to the observation of captive populations [33], in agreement with our personal observations, both sexes contribute to nest-building, incubating, and take turns feeding the young. Pair members are bonded and often clump together, even outside the breeding period.

### Subject birds

We used 29 adult Java sparrows (male: n = 14, female: n = 15; 343–639 d old) from our laboratory-bred population. Subject birds did not have prior breeding experience. To ensure reproductive motivation, all birds were kept in single-sex home cages (43 × 37 × 41 cm) for at least 1 month before the experiment started. To observe social interactions during the birds’ first encounters, we ensured that paired males and females had never directly met before.

Throughout the study, subject birds were kept in a controlled environment suitable for breeding (at a temperature of 25 ± 3°C; humidity 30–60%; 12L:12D photoperiod) and provided with finch seed mixture ad libitum, consisting of foxtail millet coated with egg yolk, rice, water, shell grit, and green vegetables. All subject birds were marked with coloured leg rings and aluminium bands for individual identification.

### Pairing test

Because testing all combinations of males and females would be difficult and could reduce reactions of subjects because of habituation to repeated encounters with different birds, we designed 56 pairing combinations, in which each male subject was tested with four females, and each female subject with two to five males, with 3–5 d between tests. To confirm that habituation in this pairing test design was minimal, we included the effect of test order (i.e., 1–4 for males, 1–5 for females) in each statistical model, as explained below. To separately test the effects of an individual’s experience and their partner’s experience, test orders of the two paired birds were not correlated.

On the day prior to the pairing test, each subject bird was isolated from its cage mates and individually introduced into an experimental cage (27 × 36 × 18 cm) equipped with a perch, and water and food cups in a soundproof room. After spending 1 night alone in the soundproof room, the female subject was moved into the male’s cage the following morning, and their behaviour was recorded with a video camera (GC-PX1, Victor, Kanagawa Japan) for 9 h (9:00–18:00). After the pairing test, each subject was returned to its home cage. All courtship episodes were extracted from the 9-h movie of each pairing test and coded (see below for the definition of courtship episode).

### Behavioural coding

The beginning of courtship was defined by either subject starting to show any courtship behaviours, including dancing (bill wiping or hopping) or singing (Fig 1). According to our preliminary observations, in the majority of the cases, courtship started with bill wiping. Bill clicking sounds were often also involved in courtship displays of both sexes [34], but were not examined in this study because bill movement associated with clicking is too subtle to identify which bird produced the sound. The courting phase was considered to be from the start of the courtship until female CSDs (Fig 1D) or until the end of courting behaviours in cases without CSDs (Fig 1E). Periods with an absence of courtship behaviours or mating events for more than 0.5 minutes were seen as separating courtship episodes.

We examined which types of behavioural interactions during the courting phase influenced mating success (i.e., the presence of CSDs, mounting, and copulation; Fig 1D and 1E). In particular, we checked whether females initiated courtship (female initiation). Though females are typically thought to be more selective than males even in monogamous species based on the frequent sexual dimorphisms in ornamental or display traits (but also see [18,34,35]), it is unclear which sex typically initiates courtship in the Java sparrow. In addition, we scored the presence/absence of duet dance, by checking whether courtship dances (i.e., bill wiping or hopping) were mutually exchanged, and examined the presence of songs in the courtship phase. Java sparrows tended to start singing by producing sparse repetition of introductory notes before the main song (Fig 1A; cf. [36]), similar to the behaviour reported in a closely related species, the Bengalese finch [37]. Presence of songs was scored as follows: 0, male produced no song during the courting phase; 1, male produced introductory notes but not the main song during the courting phase; 2, male produced at least part of the main song during the courting phase. All other behavioural variables (i.e., female initiation, duet dancing, CSD, mounting, and copulation) were scored as 1/0 (presence/absence) values.

From these data, we calculated the total number of episodes with male dance, female dance, CSD, mounting, and copulation in each male–female combination. When there were no courting behaviours (i.e., bill wiping, hopping, or singing), or mating-related behaviours (i.e., CSD, mounting, and copulation), throughout the 9-h observation period, the episode was scored zero on all measures.

### Statistical analyses

We tested whether mating success in each courtship episode was dependent on how the pair behaviourally interacted during the courting phase. In the analyses, we focused on each courtship episode, and evaluated whether mating success (i.e., as determined based on the presence of CSD, mounting, and copulation) was dependent on female initiation, and the presence of duet dancing and male song, using a generalised linear mixed model (GLMM) with binomial error distribution and a random effect of pair combination identity to account for non-independence of multiple courtship episode data from the same pair. In these analyses, both male and female experience with the pairing test was considered by entering test order (i.e., 1–4 for males, 1–5 for females) as an explanatory variable. To account for the effect of experience of some pairs that repeatedly engaged in courtship within a test, the episode order was also entered as an explanatory variable. In addition, to deal with multiple data from each pair, we extracted data from the initial courtship episode and repeated the above analyses using a GLMM with binomial error distribution with random effects of male and female identities. However, as GLMM for copulation failed to converge, we were obliged to use the GLM (without random effects).

As a supplementary analysis, we explored which factors were able to explain which sex initiated courtship in each episode. Specifically, we considered episode order, test order, and presence/absence of successful copulations in preceding episodes within pairs, and tested their effects on female initiation, using a GLMM with binomial error distribution and random effects of male and female identities. All statistical analyses were performed using R ver. 3.2.2 (R core team 2015).

## Results

We observed 240 courtship episodes in 46 male–female combinations (238 episodes included dance, and two included only song). No courtship occurred in 10 combinations. Females produced CSDs in a total of 134 courtship episodes, which was followed by mounting in 95 episodes (S2 Movie), and copulation in 54 episodes (S1 Movie). In some male–female combinations, paired birds repeatedly exhibited courtship behaviour within the 9-h observation period (0–6 CSDs/pair, 0–6 mountings/pair, 0–5 copulations/pair; S1 Fig).

### Effects of courtship interactions on mating success

**Fig 2:**
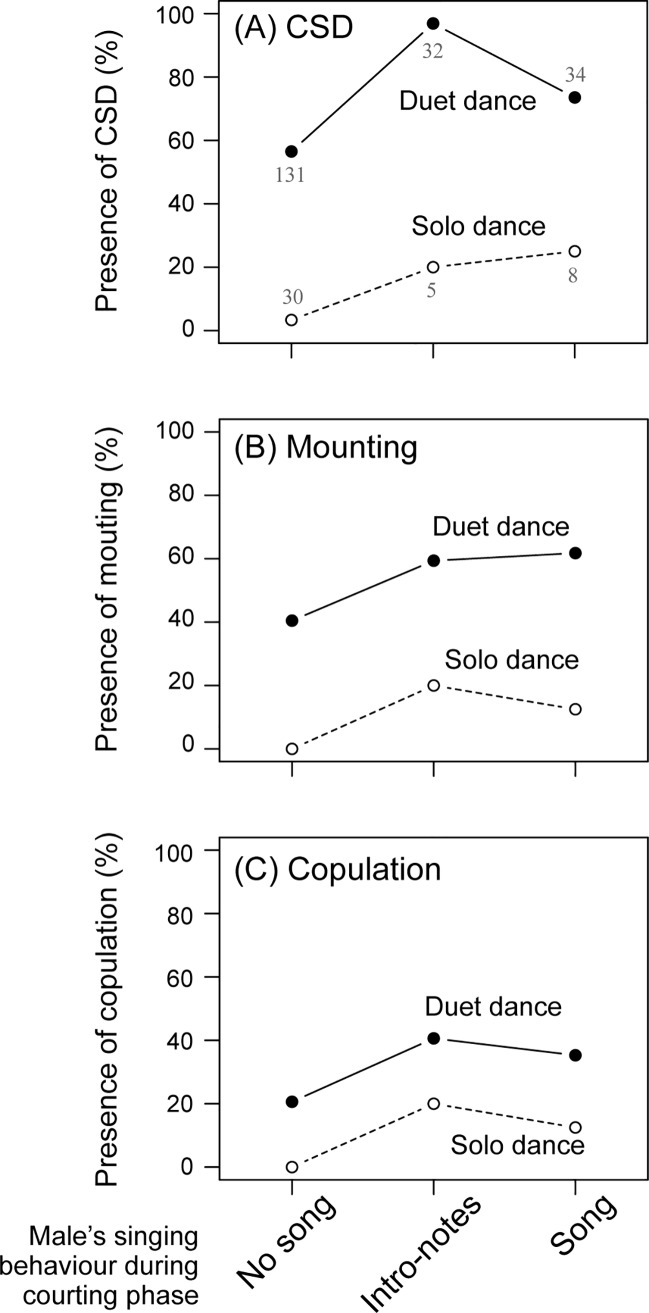
Effects of duet dance and male song on mating success. Mating success is plotted as the probability of occurrence (%) of CSD (A), mounting (B), or copulation (C) per observed episodes, depending on male’s singing behaviour during courtship phase and the presence of duet dancing. Solo dance means absence of duet dance (i.e., only one of the paired birds showed dance). Each number in (A) shows the sample size (the number of courtship episodes), which was consistent in (B) and (C).

We predicted that behavioural interactions between paired birds would be responsible for the mating success of each courtship episode. The results partially confirmed this prediction, because duet dancing and male singing contributed to higher probability of occurrence of CSD, mounting, and copulation (GLMM, p < 0.007; Table 1A, Fig 2). Importantly, we found that females frequently performed CSDs before listening to songs or when they had only heard introductory notes (Fig 2A). However, female initiation had no effect on mating success (GLMM, p > 0.87; Table 1A). In addition, although there were no habituation effects caused by repeated pairing tests; males that experienced more pairing tests were more successful in inducing CSD, and females that experienced more pairing tests were more likely to be mounted by males (Table 1A). However, there was no tendency for a greater number of courtship episodes within pairs to lead to higher mating success (Table 1A).

**Table 1:** Effects of behavioural interactions between paired birds on mating success (occurrence of CSD, mounting, and copulation) in all courtship episodes (A) and initial courtship episode of each pair (B). As the subject birds experienced multiple pairing tests with different partners, test order was included as an explanatory variable to control for it (A, B). As some pairs engaged in multiple courtships in a test, the order of courtship episodes was also included as an explanatory variable (A).

**(A) All courtship episodes**																	
			CSD					Mounting					Copulation				
Explanatory variable		(category / scale)	Coef	SE	z	p		Coef	SE	z	p		Coef	SE	z	p	
Intercept			-4.51	1.08	-4.17	< 0.001		-6.66	1.67	-3.99	< 0.001		-5.55	1.84	-3.01	0.003	
**Behavioural interaction**	Initiating sex	(**female** or male) *^1^	-0.02	0.34	-0.07	0.941		-0.12	0.41	-0.29	0.775		-0.01	0.47	-0.02	0.987	
	Dance	(**duet** or solo) *^1^	3.42	0.62	5.50	**< 0.001**	*	4.13	0.96	4.32	**< 0.001**	*	3.13	0.99	3.15	**0.002**	*
	Song	(0, 1, 2) *^2^	0.87	0.31	2.83	**0.005**	*	0.82	0.30	2.75	**0.006**	*	1.00	0.37	2.70	**0.007**	*
**Experience**	Episode order		0.02	0.06	0.30	0.762		-0.03	0.07	-0.44	0.660		-0.14	0.09	-1.45	0.146	
	Male test order		0.36	0.17	2.14	**0.032**	*	0.12	0.29	0.41	0.682		0.06	0.35	0.18	0.859	
	Female test order		0.19	0.19	1.02	0.309		0.83	0.32	2.55	**0.011**	*	0.30	0.36	0.84	0.403	
**(B) Initial courtship episodes**																	
			CSD					Mounting					Copulation				
Explanatory variable		(category / scale)	Coef	SE	z	p		Coef	SE	z	p		Coef	SE	z	p	
Intercept			-5.78	2.42	-2.39	0.017		-7.12	2.64	-2.70	0.007		-9.10	3.58	-2.54	0.011	
**Behavioural interaction**	Initiating sex	(**female** or male) *^1^	-0.91	1.01	-0.91	0.365		0.32	0.98	0.33	0.744		1.46	1.18	1.23	0.219	
	Dance	(**duet** or solo) *^1^	3.91	1.26	3.12	**0.002**	*	3.06	1.22	2.51	**0.012**	*	2.74	1.32	2.07	**0.039**	*
	Song	(0, 1, 2) *^2^	0.95	0.60	1.59	0.113		1.24	0.62	1.98	**0.047**	*	2.44	1.35	1.81	0.070	**†**
**Experience**	Male test order		0.25	0.46	0.54	0.592		0.14	0.45	0.30	0.762		0.38	0.47	0.81	0.419	
	Female test order		0.50	0.45	1.12	0.264		0.83	0.46	1.81	0.071	**†**	1.10	0.55	1.98	**0.048**	*

*1: Estimated coefficients were for the categories shown with bold (female and duet).

*2: 0, male produced no song during the courting phase; 1, male produced introductory notes but not the main song during the courting phase; 2, male produced at least part of the main song during the courting phase.

Each coefficient was estimated from GLMM or GLM with binomial error distribution, and the significance of explanatory variables is indicated with

*: p < 0.05 or

†: p < 0.1.

Although it is possible that females did not need to listen to a whole song after they had already heard it in the first courtship episode, the results from the analyses on initial episodes revealed the opposite, showing a weaker and non-significant song effect on CSD (GLMM, p > 0.11; Table 1B). However, duet dancing contributed to a higher probability of occurrence of CSD, mounting, and copulation in the first courtship episode (Table 1B), consistent with the results from all courtship episodes (Table 1A).

### Which sex initiated courtships

Dances were approximately equally likely to be initiated by females or males (125 vs 113 courtship episodes, binomial test; p = 0.437), more than 80% of which (n = 197) induced dancing of the partner bird, which was considered duet dancing in this study (S1 and S2 Movies; S2 Fig). The occurrence of duet dancing was not significantly related to female initiation (GLMM, p = 0.153), indicating that duet dancing could be triggered by both sexes.

Although we examined the effects of episode order, test order, and presence/absence of successful copulations in preceding episodes on female initiation, none of these variables had significant effects (GLMM, episode order: p = 0.903, female test order: p = 0.099, male test order: p = 0.810, experience of copulation success: p = 0.093). However, there were significant individual differences in the probability initiating courtship among males (p = 0.027) and females (p < 0.001).

## Discussion

The current study revealed that mutual courtship dancing played an essential role in mating in the Java sparrow. Importantly, this finding contrasts with the majority of past songbird research focused on vocalisations (i.e., songs) as primary behavioural sexual signals.

In the current study, we observed behavioural interactions between two opposite sex birds on first encounter, finding that mutual courtship dances (duet dances) predicted higher mating success, as determined by the occurrence of CSD, mounting, and copulation. Moreover, the results revealed that duet dancing was initiated by either sex, and initiator sex did not affect mating outcomes, supporting the view that courtship in this species is a bilateral process. Whereas past research primarily focused on the adaptive significance of mutual displays exchanged between already pair-bonded males and females [20,32,38], our findings suggest that duet dancing may also be involved in “matchmaking” by mutually confirming the willingness to mate.

Although the results revealed that songs of males also contributed to mating success (Table 1), we speculate that this was less influential than duet dancing. In many courtship episodes, females began to show CSDs before males sang, or when they had only heard introductory notes (Fig 2A), indicating that female mate decisions were made regardless of the main song. This was still true when initial courtship episodes were examined alone (Table 1B), meaning that females not knowing their partner’s song often still showed CSD following duet dancing. This finding is surprising, given that main song phrase shows great among-individual variation in acoustic complexity, and has thus traditionally been thought to be important for mate choice in some Estrildid species [39–41] (also see [10,42,43]). Although our findings appear to suggest that song is less important as a sexual signal in the Java sparrow, we propose that dance, in contrast with song, is particularly important for close-distance communication. Because Java sparrows sing undirected songs [36,44], presumably contributing to advertisement directed to potential mates or assessment of rival males, as reported in other related species (cf. zebra finch [45]), it is likely that songs provide clues for mate choice before females reach close proximity with males.

We observed repeated courtship displays on first encounter of some pairs (S1 Fig), which occurred within a relatively short time (i.e., 9-h observation of pairing test). As we tested birds that did not have prior breeding experience and were kept with same-sex birds, they may have been eagerly searching for mates. Accumulated experience of courtships within pairs did not necessarily increase mating success in later courtship episodes. However, increased experience (as test order progressed) was associated with more success in mating (Table 1). It remains unclear whether such repeated events of mutual courtship and copulation lead to pair-formation or influence reproductive performance in this species [46,47]. This issue should be investigated further in future studies.

In previous studies of various bird species, female preference for male visual displays has been explained in terms of aggressiveness or intensity (e.g., brown-headed cowbird [48], bowerbirds [49–51]), and motor performance (e.g., manikins [52,53]; reviewed in [5]). One study of the diamond firetail (*Stagonopleura guttata*; an Estrildid species that exhibits male dancing but not female dancing) reported that females preferred faster courtship displays (i.e., a faster bobbing rate) [10]. In the blue-capped cordon-bleu, an Estrildid species known for tap-dancing courtship behaviour among males and females, it remains unclear whether mating preference is based on tap speed [11]. In Java sparrows, it is difficult to determine whether particular dance features are advantageous for mating, because bill wiping and hopping rate could be condition-dependent signals of individuals, but may also be influenced by the behaviours of the partner bird (S1 and S2 Movies). Mutual dance displays are subject to synergistic effects between two birds, making it difficult to determine clear causal relationships in linked sequences of courtship dances. Moreover, the link between duet and mating outcomes does not necessarily indicate causality, if both are consequences of mutual mate choice. Careful experimental control of one of these variables will be crucial, though difficult, for investigating whether and how males and females base their mating preferences on dance.

Overall, the current study highlights the necessity of investigating temporal coordination of mutual courtship between the sexes to further determine the function of the Java sparrow’s mutual display. Because this study was, to our knowledge, the first to investigate mutual interactions of Estrildid courtship with a focus on the presence or absence of particular behaviours, we used a relatively broad definition of “duet dance”. Future studies should examine the precise temporal relationships between the courtship dances of paired birds, and whether the degree of coordination affects mating outcomes. In addition, determining the ways in which duet dancing changes over time within Java sparrow pairs may provide valuable insight, because several previous studies of vocal duets suggest that the degree of duet coordination increases with pair-bond duration in some species [22,23]. Although fine temporal coordination is known to signal the strength of a pair’s relationship to rivals [20], the finding that Java sparrows exhibited mutual duet dancing when no other individuals were present indicates that the primary function of duet dancing is for potential mating partners, not bystanders.

## Supporting information

S1 Movie Duet dancing followed by mounting and copulation. (MP4)

S2 Movie Duet dancing followed by mounting. (MP4)

S3 Movie Female solo dancing not leading to mating. (MP4)

S1 Fig Frequency distribution of the number of CSDs (a), mountings (b), and copulations (c) / pair.
(PDF)

S2 Fig Proportion of female-initiated and male-initiated courtship dances, in relation to occurrence of duet dance and CSD. (PDF)
