# Supplementary figures and images for: Mating success follows duet dancing in the Java sparrow
Source: PLoS One. 2017 Mar 8;12(3):e0172655. doi: 10.1371/journal.pone.0172655 (PMC5342200; doi:10.1371/journal.pone.0172655)

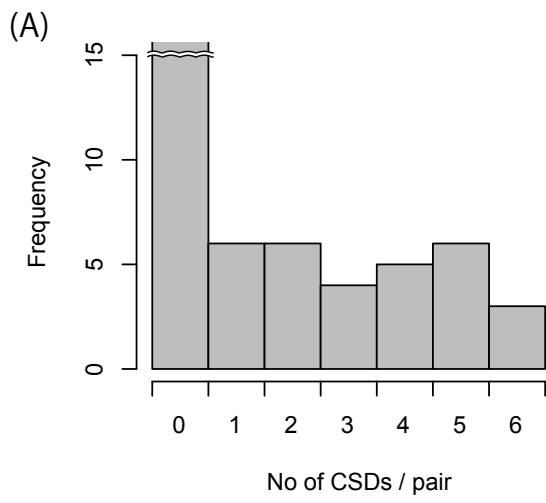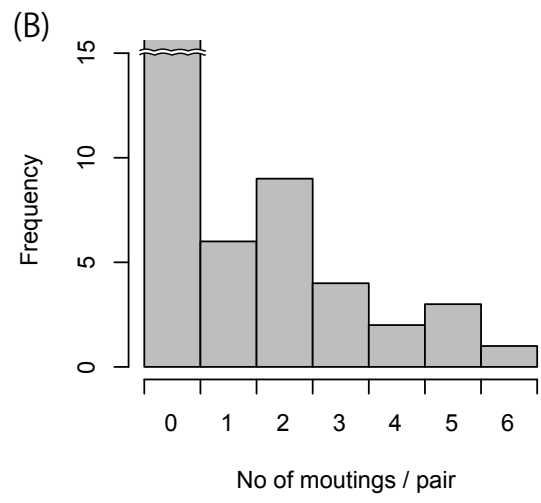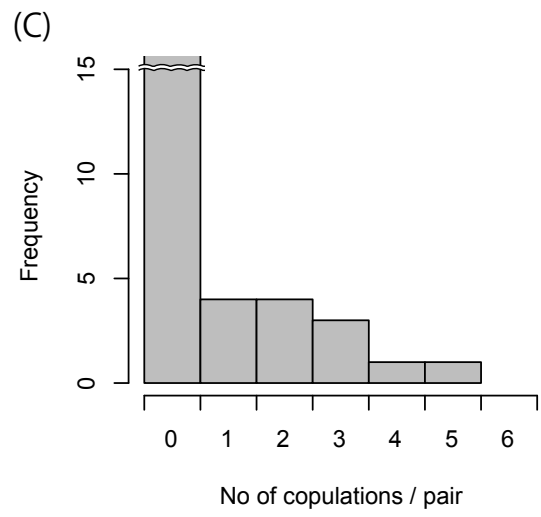

Figure S1. Frequency distribution of the number of CSDs (A), mountings (B), and copulations (C) per pair.

Supplement: S1 Fig — Frequency distribution of the number of CSDs (a), mountings (b), and copulations (c) / pair. (PDF) [file pone.0172655.s004.pdf]
