# Supplementary material for: Mating success follows duet dancing in the Java sparrow
Source: PLoS One. 2017 Mar 8;12(3):e0172655. doi: 10.1371/journal.pone.0172655 (PMC5342200; doi:10.1371/journal.pone.0172655)
Supplement: S2 Fig — (PDF) [file pone.0172655.s005.pdf]

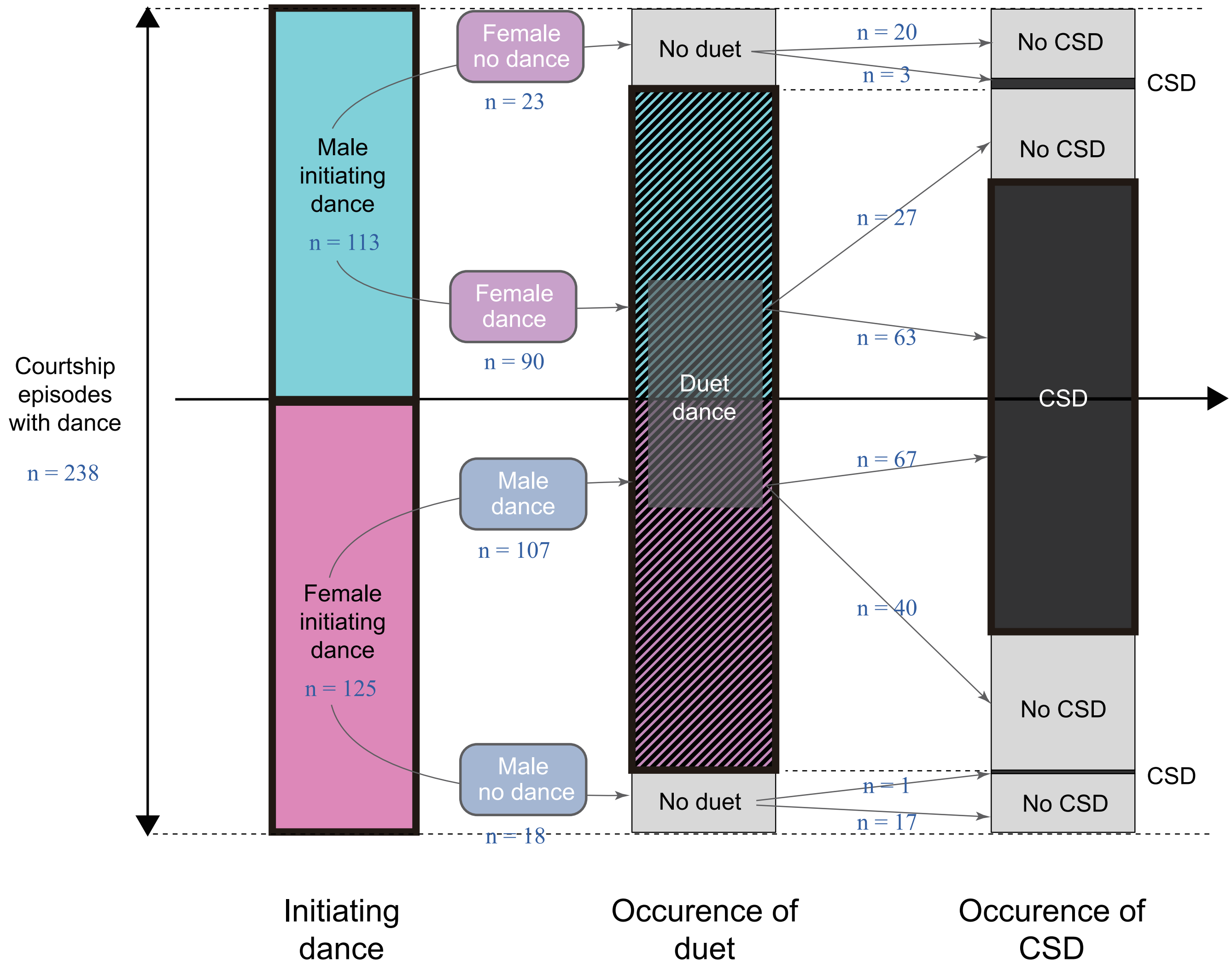

Figure S2. Proportion of female-initiated and male-initiated courtship dance, in relation to occurrence of duet dance and CSD.
